# The evidence for the use of recombinant factor VIIa in massive bleeding: revision of the transfusion policy framework

**DOI:** 10.1111/j.1365-3148.2012.01164.x

**Published:** 2012-12

**Authors:** Y Lin, C J Moltzan, D R Anderson, on behalf of the National Advisory Committee on Blood and Blood Products

**Affiliations:** 1 Department of Clinical Pathology, Sunnybrook Health Sciences CentreOntario; 2 Department of Laboratory Medicine and Pathobiology, University of TorontoOntario; 3 Department of Internal Medicine, University of ManitobaWinnipeg, Manitoba; 4 Department of Medicine, Capital Health and Dalhousie UniversityHalifax, Nova Scotia, Canada

**Keywords:** massive transfusion, off-label use, recombinant factor VIIa

## Abstract

In 2006, the Canadian National Advisory Committee on Blood and Blood Products (NAC) developed a transfusion policy framework for the use of off-label recombinant factor VIIa (rFVIIa) in massive bleeding. Because the number of randomised controlled trials has doubled, the NAC undertook a review of the policy framework in 2011. On the basis of the review of 29 randomised controlled trials, there remains little evidence to support the routine use of rFVIIa in massive bleeding. Mortality benefits have not been demonstrated. Contrarily, an increase in arterial thromboembolic events has been observed with the use of off-label rFVIIa. Given the absence of evidence of benefit and with evidence of the risk of harm, the NAC recommends that recombinant VIIa no longer be used for the off-label indications of prevention and treatment of bleeding in patients without haemophilia.

In 2008, the Canadian National Advisory Committee on Blood and Blood Products (NAC) published a policy framework for the use of recombinant factor VIIa (rFVIIa) in massive bleeding (Moltzan *et al.*, 2008). Its purpose was to provide Canadian hospitals with recommendations on the medical and prerequisite conditions for appropriate use of rFVIIa based on the existing medical literature. At that time, increasing use of off-label rFVIIa was being reported (Cameron *et al.*, 2007; Isbister *et al.*, 2008; Karkouti *et al.*, 2008) and at a cost of over CDN$1000 per mg, concerns about its costs, benefits and risks were justified. Since that time, the number of available controlled clinical trials has doubled and more data have been published on the potential risks of rFVIIa. The objective of this article is to update the policy framework for the Canadian setting by reviewing the updated literature available on the benefits and risks of rFVIIa in patients without haemophilia. Particular questions of interest are as follows: What is the current use of rFVIIa in Canada? What is the evidence for the use of off-label rFVIIa in the most commonly used clinical settings? What are the benefits of rFVIIa? What are the risks? On the basis of these updated findings, what are the NAC recommendations on the use of off-label rFVIIa in patients without haemophilia?

## BACKGROUND

The NAC is an interprovincial medical and technical advisory body to the provincial and territorial health ministries that fund the blood system in Canada. In 2006, the NAC assembled a panel of 11 experts to review the evidence from randomised clinical trials to reach consensus on the use of rFVIIa in a variety of off-label settings. The recommendations and conclusions were based on interpretation of the available evidence and where evidence was lacking, on consensus expert clinical opinion (Moltzan *et al.*, 2008).

In 2011, an update of the policy framework was planned. A review of the literature was conducted based on a search strategy published in the most recent Cochrane systematic review (Simpson *et al.*, 2012). Briefly, the search strategy included MEDLINE, EMBASE, CINAHL and CENTRAL (Cochrane Central Register of Controlled Trials, The Cochrane Library 2011, Issue 1) databases and was conducted up to March 2011. The literature search focused on controlled clinical trials on rFVIIa in patients without haemophilia. Data were summarised and presented to a panel of transfusion medicine experts on the NAC. The policy framework was revised taking into account the new data available and presented to the NAC members for feedback in November 2011. Modifications arising from the consultative process were incorporated into this document.

## TRENDS IN rFVIIa USE

Although rFVIIa was licensed in 1999 in Canada (Health Canada, 2005) and the United States (Center for Biologics Evaluation and Research, 1999) for the treatment of bleeding in haemophilia A/B patients with inhibitors, it was recognised early on that rFVIIa could be exploited for its haemostatic effect (Kenet *et al.*, 1999) and used off-label in a variety of complex clinical situations to prevent or treat significant bleeding. In fact, its use continued to grow at least until 2008 when reports showed that only 1–3% of patients being treated with rFVIIa had haemophilia (Logan *et al.*, 2011; Phillips *et al.*, 2011). In the report of American hospitals, use of off-label rFVIIa increased by 143-fold from 2000 to 2008. In 2008, the top indications for in-hospital use of rFVIIa were cardiac surgery (27%), trauma (18%) and intracranial haemorrhage (ICH; 11%) (Logan *et al.*, 2011). In the Haemostasis Registry final report of rFVIIa use in Australia and New Zealand between 2000 and 2009, rFVIIa use reached a plateau in 2006 to 2008 with a slight decline in 2009 (Phillips *et al.*, 2011). Similarly, the largest users in 2009 were cardiac surgery patients (45%), patients who had undergone ‘other surgery’ (18%) and trauma (13%).

**Fig. 1:**
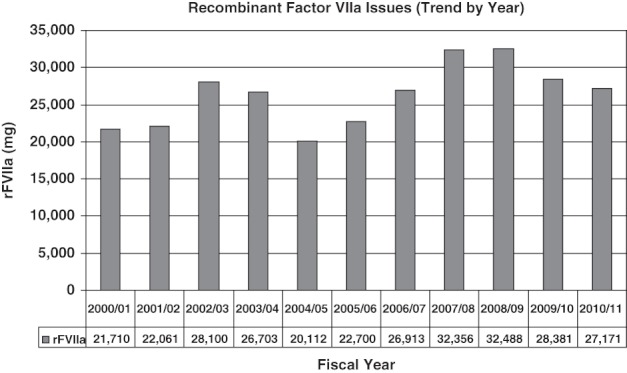
Trend in recombinant factor VIIa issues by Canadian Blood Services (courtesy of Canadian Blood Services).

In Canada, the trends in rFVIIa issues from Canadian Blood Services (the nation's sole blood supplier with the exception of the province of Québec) showed a rise until 2008 and then a slight decline in 2009 and 2010 (Fig. 1; David Howe, Executive Director, Product & Hospital Services, Canadian Blood Services, 23 September 2011, personal communication). In fact, data from a Canadian registry of off-label rFVIIa use confirms this trend (Keyvan Karkouti, Department of Anesthesia, University of Toronto, 7 November 2011, personal communication). The registry included 16 Canadian hospitals and captured data on off-label use at each site between 2007 and 2010. These 16 hospitals accounted for approximately 80% of cardiac surgery cases performed in Canada. The main indications in the off-label registry were comparable to our international counterparts with cardiac surgery (71%) being the predominant indication followed by trauma (7%), ICH (7%) and liver/abdominal surgery (4%).

## RANDOMISED TRIALS OF USE OF rFVIIa IN PATIENTS WITHOUT HAEMOPHILIA

To date, there have been 27 published randomised controlled clinical trials (RCTs) on the use of off-label rFVIIa. The studies have been conducted in a variety of settings. The following sections focus on situations where off-label rFVIIa is most commonly used in Canada and where there is more than one RCT to guide our decisions.

### Cardiac surgery

Five RCTs have been conducted in patients undergoing cardiac surgery: four RCTs where rFVIIa was given after heparin reversal post-cardiopulmonary bypass (CPB; Diprose *et al.*, 2005; Ekert *et al.*, 2006; Ma *et al.*, 2006; Essam, 2007) and one RCT in the immediate post-operative setting (Gill *et al.*, 2009). Diprose *et al.* evaluated rFVIIa in reducing transfusion and found a reduction in the need for transfusion in 20 patients undergoing complex non-coronary cardiac surgery requiring CPB. Ma *et al.* evaluated rFVIIa in 22 patients undergoing cardiac valve surgery with CPB. No specific primary outcome was stated but a reduction in transfusion was observed with rFVIIa. Essam evaluated 30 patients undergoing elective coronary revascularisation. No specific primary outcome was defined but a decrease in chest tube drainage favouring rFVIIa was noted. All three studies were small and did not show a mortality benefit. Ekert *et al.* studied 82 infants with congenital heart disease requiring CPB. The primary outcome was time to chest closure. They found prolonged time to chest closure with rFVIIa and no difference in blood loss or transfusion requirements. Finally, Gill *et al.* studied 179 patients who had undergone surgery requiring CPB but had been in the post-operative care environment for at least 30 min and had met a defined bleeding rate for rFVIIa administration. The primary outcome was serious adverse events. There were more serious adverse events in the rFVIIa group, although this was not statistically significant. Secondary outcomes including need for reoperation for bleeding and allogeneic blood transfusion favoured the rFVIIa group.

A recent systematic review did explore the role of rFVIIa in the cardiac surgery setting (Yank *et al.*, 2011). This included two RCTs (Diprose *et al.*, 2005; Gill *et al.*, 2009) as well as four observational studies. The review found that there was no mortality benefit and an increase in thromboembolic (TE) events with rFVIIa [risk difference (RD) 0·05; 95% CI: 0·01 to 0·10]. On the basis of the current available data, rFVIIa is not recommended in the treatment of patients undergoing cardiac surgery requiring CPB.

### Trauma

There have been two publications on the use of rFVIIa in trauma (Boffard *et al.*, 2005; Hauser *et al.*, 2010). Both publications reported simultaneous RCTs in a blunt and penetrating trauma population. The first publication (Boffard *et al.*, 2005) was a multicentre study on patients with severe trauma randomised after the sixth RBC unit had been administered. The primary endpoint was the number of RBC units transfused during the 48-h period after the first dose of study drug. There was no difference between the control group and rFVIIa groups for either blunt or penetrating trauma. However, when only blunt trauma patients alive at 48 h were considered, there was a statistically significant reduction of 2·6 units with the use of rFVIIa (*P* = 0·02). There were no differences in the secondary outcomes of 48-h or 30-day mortality. The second publication (Hauser *et al.*, 2010) was a phase 3 multicentre RCT in bleeding blunt and/or penetrating trauma patients. Patients were treated with study drug after receiving four units of RBCs. There was no difference in the primary outcome of 30-day mortality between the rFVIIa and placebo groups. This study was terminated early because of a lower than expected mortality rate and a high likelihood of futility in demonstrating the primary endpoint favouring rFVIIa in the blunt trauma population.

A systematic review of these two trials did not show a benefit in mortality, nor did it show an increase in TE complications (Yank *et al.*, 2011). It did, however, show a benefit favouring rFVIIa in reducing the risk of acute respiratory distress syndrome (RD −0·05; 95% confidence interval (CI): −0·02 to −0·08). Given the lack of mortality benefit, rFVIIa is not recommended in the treatment of patients with blunt or penetrating trauma.

### Spontaneous ICH

There have been four RCTs evaluating rFVIIa in spontaneous ICH administered within 3 h of symptom onset (Mayer *et al.*, 2005a, b, 2006, 2008). Two studies had primary outcomes of adverse events and found no significant differences between placebo and rFVIIa (Mayer *et al.*, 2005b, 2006). The phase II trial evaluated the percent change in ICH volume at 24 h and showed that ICH volume increased more in the placebo group than the rFVIIa group (Mayer *et al.*, 2005a). Secondary outcomes suggested that severe disability and mortality were improved in the rFVIIa group; however, the rFVIIa group also had a trend to increased risk of adverse thrombotic events. The phase 3 study on 841 patients was designed with a primary combined outcome of severe disability or death at 90 days after ICH (Mayer *et al.*, 2008). There was no difference in the combined outcome between the rFVIIa treated groups compared with placebo and in fact, a statistically significant increase in arterial events was observed in the group receiving 80 mcg kg^−1^ rFVIIa compared with placebo. It should be noted that there are two ongoing RCTs (initiated in 2010 and 2011) examining the role of a single dose of 80 mcg kg^−1^ rFVIIa in a subgroup of patients with acute ICH who have a ‘spot sign’ on computed tomography (CT) angiography indicating contrast extravasation and active bleeding (Flaherty & Jauch, 2011; Gladstone *et al.*, 2011). The estimated dates of completion for these studies are 2013 and 2015, respectively.

A systematic review of the ICH RCTs and one observational trial did not show benefit in mortality or poor functional outcome with the use of rFVIIa (Yank *et al.*, 2011). However, an increase in arterial thromboembolism was seen with rFVIIa for medium (>40 but <120 mcg kg^−1^) and high (≥120 mcg kg^−1^) doses (medium RD 0·03, 95% CI: 0·01 to 0·06; high RD 0·06, 95% CI: 0·01 to 0·11). As a mortality benefit has not been shown and there is increased risk of arterial thromboembolism, rFVIIa is not recommended for the treatment of ICH.

### Liver transplantation

Three RCTs evaluated the effect of rFVIIa in orthotopic liver transplantation (OLT). The two larger studies (209 and 87 patients, respectively) had a primary outcome of transfusion requirements and neither study showed a difference between placebo and rFVIIa groups (Lodge *et al.*, 2005a; Planinsic *et al.*, 2005). The third study consisting of 20 patients did not state a primary outcome but found a decrease in INR, blood loss and transfusion requirements (Pugliese *et al.*, 2007). No mortality benefit was seen in any of the studies. rFVIIa use in the treatment of patients undergoing OLT is not recommended.

### Liver resection

Two RCTs evaluated the effect and safety of rFVIIa in partial hepatectomy (Lodge *et al.*, 2005b; Shao *et al.*, 2006). Both studies had a primary outcome of transfusion requirements and neither showed a difference in the required RBC units between placebo and rFVIIa groups. rFVIIa use in the treatment of patients undergoing surgery for liver resection is not recommended.

### Cirrhosis with gastrointestinal bleeding

Two RCTs evaluated rFVIIa in controlling upper gastrointestinal bleeding (UGIB) in patients with cirrhosis when administered as an add-on to standard therapy (Bosch *et al.*, 2004, 2008). The primary outcome in both studies was failure to control bleeding. In the initial RCT, there was no difference observed (Bosch *et al.*, 2004). However, a subgroup analysis suggested that there might be benefit of using rFVIIa in patients with more advanced liver disease (Child Pugh class B and C). The second RCT specifically included Child Pugh class B and C patients with cirrhosis and variceal bleeding (Bosch *et al.*, 2008). Again, there was no difference in the primary outcome of failure to control bleeding. rFVIIa use in the treatment of UGIB in patients with cirrhosis is not recommended.

### Other settings

In addition to the RCTs outlined in the previous sections, off-label rFVIIa has also been studied in liver biopsy (Jeffers *et al.*, 2002), prostatectomy (Friederich *et al.*, 2003), pelvic fracture (Raobaikady *et al.*, 2005), haematopoietic stem cell transplant (Pihusch *et al.*, 2005), dengue haemorrhagic fever (Chuansumrit *et al.*, 2005), burn patients requiring surgery (Johansson *et al.*, 2007), spinal fusion surgery (Sachs *et al.*, 2007), traumatic ICH (Narayan *et al.*, 2008) and craniofacial reconstruction (Hanna *et al.*, 2010). All these studies were small with 100 patients or less and only four had more than 50 patients (Jeffers *et al.*, 2002; Pihusch *et al.*, 2005; Sachs *et al.*, 2007; Narayan *et al.*, 2008). No mortality benefit was seen in any of these studies. The effect of rFVIIa on the primary outcome of these RCTs is summarised in Tables 1 (prophylactic RCTs where rFVIIa was used to prevent bleeding) and 2 (therapeutic RCTs where rVIIa was used to treat bleeding). Given the limited data, these studies do not demonstrate sufficient evidence to recommend the use of rFVIIa in any of these indications.

**Table 1:** Summary of prophylactic randomised controlled trials using off-label rFVIIa

Category	Study	Participants	Number of patients (rFVIIa/C)	Dosing	Primary outcome	Primary results
Cardiac surgery	Diprose *et al.* (2005)	Complex non-coronary cardiac surgery requiring CPB	20 (10/10)	1 dose 90 mcg kg^−1^ or placebo after CPB	Number of patients receiving allogeneic transfusion (total); total units of RBC and coagulation products; occurrence of AE	Number of patients transfused: rFVIIa 2 vs C 8 patients (*P* = 0·037)
						Units transfused: rFVIIa 13 vs C 105 units (*P* = 0·011)
						No difference in AE
	Ekert *et al.* (2006)	Infants <1 year with congenital heart disease undergoing cardiac surgery requiring CPB	82 (36/40)	40 mcg kg^−1^ or placebo after CPB; repeated up to two times if ongoing bleeding	Time to chest closure after reversal of heparin	rFVIIa 99 min vs C 55 min (*P* = 0·0263)
	Ma *et al.* (2006)	Cardiac valve replacement surgery requiring CPB	22 (11/11)	1 dose 40 mcg kg^−1^ or placebo after CPB	No stated primary outcome	No stated primary outcome but reported decreased blood loss and transfusion requirements in rFVIIa group
	Essam (2007)	Elective cardiac revascularisation requiring CPB	30 (15/15)	1 dose 90 mcg kg^−1^ after CPB (control: no rFVIIa)	No stated primary outcome	No stated primary outcome but reported decreased blood loss and transfusion requirements in rFVIIa group
	Gill *et al.* (2009)	Cardiac surgery requiring CPB and admitted to post-operative care environment for at least 30 min	179 (104/68)	A single dose 40, 80 mcg kg^−1^ or placebo on reaching pre-defined bleeding trigger	Critical serious AE at 30 days	No difference: rFVIIa 80 mcg kg^−1^, 12% vs 40 mcg kg^−1^, 14% vs placebo 7%
Hepatic procedures	Lodge *et al.* (2005a, b)	Liver transplantation	209 (121/61)	First dose 60, 120 mcg kg^−1^ or placebo; repeated every 2 h until end of surgery	Units of RBC transfused during the perioperative period (24 h)	No difference: rFVIIa 60 mcg kg^−1^, 7·0 vs 120 mcg kg^−1^, 6·3 vs C 8·2 units
	Planinsic *et al.* (2005)	Liver transplantation	87 (54/19)	A single dose 20, 40, 80 mcg kg^−1^ or placebo	Units of RBC transfused during the perioperative period (24 h)	No difference: rFVIIa 20 mcg kg^−1^, 10·0 vs 40 mcg kg^−1^, 13·0 vs 80 mcg kg^−1^, 10·0 vs C 11·1 units
	Pugliese *et al.* (2007)	Liver transplantation	20 (10/10)	1 dose 40 mcg kg^−1^ or placebo	No stated primary outcome	No stated primary outcome but reported decreased blood loss and transfusion requirements in rFVIIa group
	Lodge *et al.* (2005a, b)	Non-cirrhotic patients undergoing partial hepatectomy	204 (112/63)	First dose 20, 80 mcg kg^−1^ or placebo; second dose at 5 h if operation expected to be longer than 6 h	Number of patients receiving allogeneic transfusion during perioperative period (48 h)	No difference: rFVIIa 20 mcg kg^−1^, 41% vs 80 mcg kg^−1^, 25% vs C 37% (*P* = 0·09)
	Shao *et al.* (2006)	Cirrhotic patients undergoing partial hepatectomy	235 (145/76)	First dose of 50, 100 mcg kg^−1^ or placebo; repeated every 2 h until end of surgery (maximum four doses)	Number of patients receiving allogeneic transfusion and units of RBC transfused during perioperative period (48 h)	No difference in either outcome. Patients transfused: rFVIIa 50 mcg kg^−1^, 51% vs 100 mcg kg^−1^, 36% vs C 38% (*P* = 0·59)
						Units transfused: rFVIIa 50 mcg kg^−1^, 0·9 vs 100 mcg kg^−1^, 0 vs C 0 units (*P* = 0·68)
	Jeffers *et al.* (2002)	Cirrhosis and coagulopathy undergoing laparoscopic liver biopsy	66 (66/0)	A single dose 5, 20, 80 or 120 mcg kg^−1^ (no control group)	Duration of normal PT	Longer duration of normal PT with higher doses of rFVIIa (80, 120 mcg kg^−1^) vs lower doses (5, 20 mcg kg^−1^)
Other surgical settings	Friederich *et al.* (2003)	Retropubic prostatectomy	36 (24/12)	A single dose 20, 40 mcg kg^−1^ or placebo	Blood loss during perioperative period (24 h) and units of RBC transfused	Blood loss: rFVIIa 20 mcg kg^−1^, 1235 mL vs 40 mcg kg^−1^, 1089 mL vs C 2688 mL (*P* = 0·001)
						Units transfused: rFVIIa 20 mcg kg^−1^, 0·6 vs 40 mcg kg^−1^, 0 vs C 1·5 units (*P* = 0·0003)
	Raobaikady *et al.* (2005)	Reconstructive surgery for traumatic pelvic fractures	48 (24/24)	First dose 90 mcg kg^−1^ or placebo; second dose at 2 h if ongoing bleeding	Blood loss during perioperative period (48 h)	No difference: rFVIIa 2070 mL vs C 1535 mL (*P* = 0·79)
	Johansson *et al.* (2007)	Thermal burns undergoing skin excision and grafting	18 (9/9)	First dose 40 mcg kg^−1^ or placebo; second dose at 90 min	Units of blood components transfused per patient and percentage full thickness wound in perioperative period (24 h)	rFVIIa 0·9 vs C 2·2 units (*P* = 0·0013)
	Sachs *et al.* (2007)	Spinal fusion surgery	60 (36/13)	First dose 30, 60, 120 mcg kg^−1^ or placebo; given at dosing trigger and repeated at 2 and 4 h	AE at 30 days and blood loss	No difference in AE and mean surgical blood loss. However, *a priori* planned adjusted blood loss: rFVIIa 30 mcg kg^−1^, 1120 mL vs 60 mcg kg^−1^, 400 mL vs 120 mcg kg^−1^, 823 mL vs C 2536 mL (*P*≤ 0·001)
	Hanna *et al.* (2010)	Children undergoing craniofacial reconstruction surgery	45 (15/15; 15 received tranexamic acid)	100 mcg kg^−1^ bolus followed by 10 mcg kg^−1^ h^−1^ infusion until skin closure or placebo	No stated primary outcome	No stated primary outcome but reported decreased blood loss and transfusion requirements in rFVIIa group

AE, adverse events; C, control; CPB, cardiopulmonary bypass.

**Table 2:** Summary of therapeutic randomised controlled trials using off-label rFVIIa

Category	Study	Participants	Number of patients (rFVIIa/C)	Dosing	Primary outcome	Primary results
Trauma	Boffard *et al.* (2005)	Blunt trauma	158 (69/74)	200 mcg kg^−1^ followed by 100 mcg kg^−1^ at 1 and 3 h or placebo	Units of RBCs transfused within 48 h of first dose of rFVIIa	No difference: rFVIIa 7·8 vs C 7·2 units (*P* = 0·07)
						When only patients alive at 48 h included: rFVIIa 7·0 vs C 7·5 units with an estimated RBC reduction of 2·6 units favouring rFVIIa (*P* = 0·02)
	Boffard *et al.* (2005)	Penetrating trauma	143 (70/64)	200 mcg kg^−1^ followed by 100 mcg kg^−1^ at 1 and 3 h or placebo	Units of RBCs transfused within 48 h of first dose of rFVIIa	No difference: rFVIIa 4·0 vs C 4·8 units (*P* = 0·24)
						No difference for only patients alive at 48 h
	Hauser *et al.* (2010)	Blunt trauma	481 (226/255)	200 mcg kg^−1^ followed by 100 mcg kg^−1^ at 1 and 3 h or placebo	30-day mortality	Terminated early
						No difference: rFVIIa 11·0% vs C 10·7% (*P* = 0·93)
	Hauser *et al.* (2010)	Penetrating trauma	92 (47/45)	200 mcg kg^−1^ followed by 100 mcg kg^−1^ at 1 and 3 h or placebo	30-day mortality	Terminated early
						No difference: rFVIIa 18·2% vs C 13·2% (*P* = 0·40)
ICH	Mayer *et al.* (2005a, b)	Spontaneous ICH	48 (36/12)	A single dose 10, 20, 40, 80, 120, 160 mcg kg^−1^ or placebo	AE at 90 days	No difference in type, severity or frequency of AEs
	Mayer *et al.* (2006)	Spontaneous ICH	41 (32/8)	A single dose 5, 20, 40, 80 mcg kg^−1^ or placebo	AE at 90 days	No difference in type, severity or frequency of AEs
	Mayer *et al.* (2005a, b)	Spontaneous ICH	400 (303/96)	A single dose 40, 80, 160 mcg kg^−1^ or placebo	Percent change in volume of ICH at 24 h	rFVIIa 40 mcg kg^−1^, 16% vs 80 mcg kg^−1^, 14% vs 160 mcg kg^−1^, 11% vs C 29% (*P* = 0·01 rFVIIa groups combined vs placebo)
						Secondary outcomes showed reduced mortality and improved functional outcomes at 90 days with non-significant increase in thromboembolic AE
	Mayer *et al.* (2008)	Spontaneous ICH	841 (558/263)	A single dose 20, 80 mcg kg^−1^ or placebo	Combined outcome of severe disability or death at 90 days	No difference: rFVIIa 20 mcg kg^−1^, 26% vs 80 mcg kg^−1^, 29% vs C 24%
						Increased frequency of arterial thromboembolic AE in the 80 mcg kg^−1^ group (9% vs C 4%, *P* = 0·04)
	Narayan *et al.* (2008)	Traumatic ICH	97 (61/36)	A single dose 40, 80, 120, 160, 200 mcg kg^−1^ or placebo	AE at 15 days	No difference in number or type of AE
GI bleeding	Bosch *et al.* (2004)	Upper gastrointestinal haemorrhage in patients with cirrhosis	245 (121/121)	First dose of 100 mcg kg^−1^ or placebo; repeated doses at 2, 4, 6, 12, 18, 24 and 30 h	Combined endpoint of failure to control bleeding or failure to prevent rebleeding or death	No difference: rFVIIa 14% vs C 16% (*P* = 0·72). Exploratory analyses of patients with Child Pugh class B or C showed benefit with rFVIIa
	Bosch *et al.* (2008)	Upper gastrointestinal haemorrhage in patients with cirrhosis and severe liver disease (Child Pugh class B and C)	265 (170/86)	First dose 200 mcg kg^−1^ or placebo; repeated at 2, 8, 14 and 20 h (600 mcg kg^−1^ total); or repeated only at 2 h (300 mcg kg^−1^)	Combined endpoint of failure to control bleeding or failure to prevent rebleeding or death	No difference: rFVIIa 600 mcg kg^−1^, 20% vs 300 mcg kg^−1^, 13% vs C 23% (*P* = 0·37)
Other settings	Pihusch *et al.* (2005)	Bleeding post-haematopoietic stem cell transplantation	100 (77/23)	First dose of 40, 80, 160 mcg kg^−1^ or placebo; repeated every 6 h × 6	Change in bleeding score at 38 h compared with baseline	No difference
	Chuansumrit *et al.* (2005)	Children with dengue haemorrhagic fever	28 (18/10)	First dose 100 mcg kg^−1^ or placebo; second dose at 30 min if ongoing bleeding	Change in bleeding	Cessation of bleeding at 2 h: rFVIIa 75% vs C 44% (no statistics provided)

AE, adverse events; C, control; GI, gastrointestinal; ICH, intracranial haemorrhage.

## SUMMARY OF BENEFITS OF rFVIIa

The benefits of rFVIIa can be considered across the clinical trials that have been conducted. The recently updated Cochrane systematic review (Table 3) included RCTs of off-label rFVIIa and divided RCTs into prophylactic and therapeutic studies (Simpson *et al.*, 2012). There were 16 prophylactic RCTs where rFVIIa was used to prevent bleeding (Jeffers *et al.*, 2002; Friederich *et al.*, 2003; Diprose *et al.*, 2005; Lodge *et al.*, 2005a,b; Planinsic *et al.*, 2005; Raobaikady *et al.*, 2005; Ekert *et al.*, 2006; Ma *et al.*, 2006; Shao *et al.*, 2006; Essam, 2007; Johansson *et al.*, 2007; Pugliese *et al.*, 2007; Sachs *et al.*, 2007; Gill *et al.*, 2009; Hanna *et al.*, 2010). All these studies were perioperative giving rFVIIa either before the procedure or at a distinct bleeding trigger in the perioperative setting (Sachs *et al.*, 2007; Gill *et al.*, 2009). Thirteen RCTs were considered therapeutic where rFVIIa was administered to bleeding patients (Bosch *et al.*, 2004, 2008; Boffard *et al.*, 2005; Chuansumrit *et al.*, 2005; Mayer *et al.*, 2005a, b, 2006, 2008; Pihusch *et al.*, 2005; Narayan *et al.*, 2008; Hauser *et al.*, 2010). Each of the trauma publications were considered as two RCTs reporting results of a blunt and penetrating trauma population within a single publication (Boffard *et al.*, 2005; Hauser *et al.*, 2010). The results are summarised in Table 2. In the prophylactic studies, there was no difference in mortality. A non-significant trend was noted towards a decreased number of patients transfused with the use of rFVIIa. However, when volumes of total blood loss and RBCs transfused were considered, only modest differences (approximating one RBC unit) favouring rFVIIa were noted. It is important to emphasise that these results likely overestimate the effect of rFVIIa because (i) smaller studies that showed larger benefit were heavily weighted because of very precise estimation of blood loss and (ii) results from larger negative RCTs were not reported in such a manner (i.e. means and standard deviations or medians and interquartile ranges) so that they could be included in the pooled estimates. For therapeutic studies, there was no difference in mortality, control of bleeding or amount of red blood cells transfused. There was a trend to decreased number of patients transfused but this was based on a pooled estimate from only three RCTs. Although no significant increase in TE events was observed in the prophylactic or therapeutic RCTs, when TE events were combined across all 29 trials, there was a significant increase in arterial TE events (relative risk 1·45, 95% CI: 1·02 to 2·05). Overall, the review concluded that based on the available RCTs, there was little evidence of benefit for the use of off-label rFVIIa in patients without haemophilia.

**Table 3:** Summary estimates from 2012 Cochrane systematic review on rFVIIa in patients without haemophilia

Outcome	Number of studies	Number of patients	Summary estimate (95% CI)	*I*^2^ value (%)
Prophylactic use				
Mortality, RR	15	1219	1·04 (0·55 to 1·97)	0
Blood loss^1^, mL, WMD	10	707	−297 (−416 to −177)	79
Red blood cell transfusion^1^, mL, WMD	12	774	−261 (−367 to −154)	62
Number of patients receiving transfusion, RR	8	868	0·85 (0·72 to 1·01)	57
Thromboembolic events, RR	13	1159	1·35 (0·82 to 2·25)	0
Therapeutic use				
Mortality, RR	13	2856	0·91 (0·78 to 1·06)	0
Control of bleeding, RR	4	616	0·95 (0·88 to 1·03)	0
Red blood cell transfusion^1^, mL, WMD	5	911	−89 (−264 to 87)	16
Number of patients receiving transfusion, RR	3	579	0·94 (0·89 to 1·00)	0
Thromboembolic events, RR	13	2873	1·14 (0·89 to 1·47)	0
All studies				
Total thromboembolic events, RR	26	4032	1·18 (0·94 to 1·48)	0
Arterial thromboembolic events, RR	25	3849	1·45 (1·02 to 2·05)	0
Venous thromboembolic events, RR	25	3849	0·92 (0·67 to 1·26)	0

CI, confidence interval; RR, relative risk; WMD, weighted mean difference.

1 One unit of red blood cells was assumed to have a volume of 300 mL

From the available RCT data, there is little evidence of a mortality benefit of off-label rFVIIa. No single trial showed a mortality benefit with the exception of the phase 2 RCT in ICH where mortality was a secondary outcome (Mayer *et al.*, 2005a); the larger phase 3 RCT in ICH powered to detect a mortality difference was negative (Mayer *et al.*, 2008). Other signals support this conclusion. Improved outcomes with higher doses of rFVIIa were not observed in either of the recent systematic reviews described above (Yank *et al.*, 2011; Simpson *et al.*, 2012). Follow-up larger RCTs did not reinforce earlier promising results specifically in the setting of trauma (Hauser *et al.*, 2010), ICH (Mayer *et al.*, 2008) and gastrointestinal bleeding in cirrhosis (Bosch *et al.*, 2008). Even for the outcomes of blood loss and red cell transfusion requirements, the amount saved was modest and likely overestimated.

It is important to note, however, that the use of rFVIIa in the RCTs reported may differ from situations where rFVIIa may be used as a ‘last ditch’ effort or in what could be termed as ‘refractory’ bleeding. These are situations where its efficacy has not been formally assessed such as in the setting of massive bleeding in obstetrical bleeding or in the management of life-threatening bleeding associated with new oral anticoagulants such as direct thrombin inhibitors or factor Xa inhibitors. These patients with ‘refractory’ bleeding are not the patients included in the RCTs conducted to date where typically rFVIIa was used in anticipation of bleeding or at a defined point earlier in the resuscitation of a bleeding patient. Patients with refractory bleeding are more likely to be found in observational reports and registries where no well-matched control group is available for comparison, nor a group using an alternative intervention (e.g. tranexamic acid or fibrinogen concentrates). These observational reports are prone to patient selection bias and observer bias as the treatment is not masked and lack generalisability (Dzik, 2006). Interpretation of the effect of rFVIIa in refractory bleeding is further hampered by the complex coagulopathy that exists in these situations and the multiple blood components, blood products and potentially haemostatic drugs administered concurrently with rFVIIa. On the other hand, the haemostatic effect of rFVIIa may be negated by physiologic conditions such as hypothermia and acidosis (Meng *et al.*, 2003), which have been shown to adversely affect rFVIIa's effect. Registry data have reinforced the lack of benefit of rFVIIa under acidotic conditions (Isbister *et al.*, 2008; Karkouti *et al.*, 2008; Phillips *et al.*, 2011). A discussion of the management of massive bleeding is outside the scope of this review; however, a recent consensus conference statement on massive transfusion may provide additional guidance to hospitals on this challenging topic (Dzik *et al.*, 2011).

## RISKS OF rFVIIa

Because rFVIIa is a procoagulant agent, the primary concern about its toxicity is the risk of TE events. Both the Cochrane review (Simpson *et al.*, 2012) and the review by Yank *et al.* (2011) reported increased risks of arterial TE events as described above. The most comprehensive review to date specifically examining the safety of off-label rFVIIa was conducted by Levi *et al.* (2010) on data from 26 RCTs involving patients. The overall rate of arterial TE events was higher with the use of rFVIIa compared with placebo (5·5 vs 3·2%, *P* = 0·003), specifically there was an increase observed in coronary events (2·9 vs 1·1%, *P* = 0·002). No difference was observed in venous TE events (5·3 vs 5·7%). A striking finding was the increase in risk of arterial TE events with increasing age. Compared with patients of <18 years of age, a higher risk of TE events were observed in patients aged 65–74 years (odds ratio 2·11; 95% CI: 0·95 to 4·71) and even higher for those ≥75 years (odds ratio 3·02, 95% CI: 1·22 to 7·48). Similar to the Yank *et al.* review, the risk of arterial TE events increased with dose in the setting of ICH.

The risks of TE events reported in clinical trials may underestimate the true risk of TE events with rFVIIa. The RCTs included in these reviews (i) did not actively screen for TE events, (ii) excluded patients who had a recent history of TE events and (iii) may have limitations in generalisability given that only a minority of patients who were screened were enrolled into the trials. Translating these risks to the real world was raised as a concern by O'Connell *et al.* (2006), who reported on the serious TE events associated with rFVIIa reported to the United States Food and Drug Administration (FDA) Adverse Event Reporting System. Three important observations were made: (i) 90% of reports occurred when rFVIIa was used outside of its labelled indication, (ii) 65% of the reports occurred in patients outside of clinical trials and (iii) 38% of reports occurred in the setting of concomitant haemostatic agents, including blood products (plasma, platelets and cryoprecipitate) and antifibrinolytics. Although these findings are limited by passive surveillance, these observations do suggest that the risk of thromboembolism reported in RCTs may underestimate risks in the real world where sicker patients are treated with off-label rFVIIa in situations where other procoagulant blood products or drugs are also being used.

## NAC RECOMMENDATIONS ON THE USE OF OFF-LABEL rFVIIa

Given the absence of evidence of benefit and with evidence of the risk of harm, the NAC recommends that rFVIIa no longer be used for the off-label indications of prevention and treatment of bleeding in patients without haemophilia. The NAC recognises that ongoing clinical trials are evaluating rFVIIa and that this recommendation will be reconsidered if favourable findings of clinically important benefits outweighing the risks are observed.

## CONCLUSION

Current available evidence does not support the use of off-label rFVIIa for massive bleeding. Mortality benefits have not been demonstrated in randomised controlled trials. Other benefits such as reduced transfusion or blood loss are modest and may be overestimated. Contrarily, an increase in arterial TE events has been observed. Given the absence of evidence of benefit and with evidence of the risk of harm, the NAC recommends that rFVIIa no longer be used for the off-label indications of prevention and treatment of bleeding in patients without haemophilia.

## APPENDIX

The members of the National Advisory Committee on Blood and Blood Products are Lucinda Whitman (Chair, Newfoundland), David Anderson (Nova Scotia), Ted Alport (Saskatchewan), Jeannie Callum (Ontario), Karen Dallas (Saskatchewan), Dana Devine (Canadian Blood Services), Cheryl Doncaster (NAC), Jennifer Fesser (Prince Edward Island), David Howe (Canadian Blood Services), Elenore Kingsbury (Canadian Blood Services), Debra Lane (Manitoba), Vincent Laroche (Québec), Doug Morrison (British Columbia), Brian Muirhead (Manitoba), Susan Nahirniak (Alberta), Katerina Pavenski (Ontario), Tanya Petraszko (British Columbia), Lakshmi Rajappannair (New Brunswick) and Meer-Taher Shabani-Rad (Alberta).

## References

[b1] Boffard KD, Riou B, Warren B (2005). Recombinant factor VIIa as adjunctive therapy for bleeding control in severely injured trauma patients: two parallel randomized, placebo-controlled, double-blind clinical trials. The Journal of Trauma.

[b2] Bosch J, Thabut D, Albillos A (2008). Recombinant factor VIIa for variceal bleeding in patients with advanced cirrhosis: a randomized, controlled trial. Hepatology (Baltimore, Md.).

[b3] Bosch J, Thabut D, Bendtsen F (2004). Recombinant factor VIIa for upper gastrointestinal bleeding in patients with cirrhosis: a randomized, double-blind trial. Gastroenterology.

[b4] Cameron P, Phillips L, Balogh Z, Joseph A, Pearce A, Parr M, Jankelowitz G (2007). The use of recombinant activated factor VII in trauma patients: experience from the Australian and New Zealand haemostasis registry. Injury.

[b5] Center for Biologics Evaluation and Research, US Food and Drug Administration (1999). http://www.fda.gov/BiologicsBloodVaccines/BloodBloodProducts/ApprovedProducts/LicensedProductsBLAs/FractionatedPlasmaProducts/ucm089228.htm.

[b6] Chuansumrit A, Wangruangsatid S, Lektrakul Y, Chua MN, Zeta Capeding MR, Bech OM, Dengue Study Group (2005). Control of bleeding in children with dengue hemorrhagic fever using recombinant activated factor VII: a randomized, double-blind, placebo-controlled study. Blood Coagulation & Fibrinolysis: An International Journal in Haemostasis and Thrombosis.

[b7] Diprose P, Herbertson MJ, O'Shaughnessy D, Gill RS (2005). Activated recombinant factor VII after cardiopulmonary bypass reduces allogeneic transfusion in complex non-coronary cardiac surgery: randomized double-blind placebo-controlled pilot study. British Journal of Anaesthesia.

[b8] Dzik WH (2006). Off-label reports of new biologics: exciting new therapy or dubious research? Examples from recombinant activated factor VII. Journal of Intensive Care Medicine.

[b9] Dzik WH, Blajchman MA, Fergusson D (2011). Clinical review: Canadian National Advisory Committee on Blood and Blood Products – Massive Transfusion Consensus Conference 2011: Report of the panel. Critical Care.

[b10] Ekert H, Brizard C, Eyers R, Cochrane A, Henning R (2006). Elective administration in infants of low-dose recombinant activated factor VII (rFVIIa) in cardiopulmonary bypass surgery for congenital heart disease does not shorten time to chest closure or reduce blood loss and need for transfusions: a randomized, double-blind, parallel group, placebo-controlled study of rFVIIa and standard haemostatic replacement therapy versus standard haemostatic replacement therapy. Blood Coagulation & Fibrinolysis: An International Journal in Haemostasis and Thrombosis.

[b11] Essam MA (2007). Prophylactic administration of recombinant activated factor VII in coronary revascularization surgery. Internet Journal of Anesthesiology.

[b12] Flaherty ML, Jauch EC (2011). http://www.clinicaltrials.gov/ct2/show/NCT00810888.

[b13] Friederich PW, Henny CP, Messelink EJ, Geerdink MG, Keller T, Kurth KH, Buller HR, Levi M (2003). Effect of recombinant activated factor VII on perioperative blood loss in patients undergoing retropubic prostatectomy: a double-blind placebo-controlled randomised trial. Lancet.

[b14] Gill R, Herbertson M, Vuylsteke A (2009). Safety and efficacy of recombinant activated factor VII: a randomized placebo-controlled trial in the setting of bleeding after cardiac surgery. Circulation.

[b15] Gladstone DJ, Aviv RI, Demchuk AM (2011). http://www.clinicaltrials.gov/ct2/show/NCT01359202.

[b16] Hanna MG, Refaie A, Gouda N, Obaya G (2010). Reduction of peri-operative bleeding in craniofacial surgeries in pediatrics: comparison between recombinant factor VII and tranexamic acid. Egyptian Journal of Anaesthesia.

[b17] Hauser CJ, Boffard K, Dutton R (2010). Results of the CONTROL trial: efficacy and safety of recombinant activated factor VII in the management of refractory traumatic hemorrhage. The Journal of Trauma.

[b18] Health Canada (2005). http://www.hc-sc.gc.ca/dhp-mps/prodpharma/notices-avis/conditions/index-eng.php.

[b19] Isbister J, Phillips L, Dunkley S, Jankelowitz G, McNeil J, Cameron P (2008). Recombinant activated factor VII in critical bleeding: experience from the Australian and New Zealand haemostasis register. Internal Medicine Journal.

[b20] Jeffers L, Chalasani N, Balart L, Pyrsopoulos N, Erhardtsen E (2002). Safety and efficacy of recombinant factor VIIa in patients with liver disease undergoing laparoscopic liver biopsy. Gastroenterology.

[b21] Johansson PI, Eriksen K, Nielsen SL, Rojkjaer R, Alsbjorn B (2007). Recombinant FVIIa decreases perioperative blood transfusion requirement in burn patients undergoing excision and skin grafting – results of a single centre pilot study. Burns : Journal of the International Society for Burn Injuries.

[b22] Karkouti K, Beattie WS, Arellano R (2008). Comprehensive Canadian review of the off-label use of recombinant activated factor VII in cardiac surgery. Circulation.

[b23] Kenet G, Walden R, Eldad A, Martinowitz U (1999). Treatment of traumatic bleeding with recombinant factor VIIa. Lancet.

[b24] Levi M, Levy JH, Andersen HF, Truloff D (2010). Safety of recombinant activated factor VII in randomized clinical trials. The New England Journal of Medicine.

[b25] Lodge JP, Jonas S, Jones RM (2005a). Efficacy and safety of repeated perioperative doses of recombinant factor VIIa in liver transplantation. Liver Transplantation: Official Publication of the American Association for the Study of Liver Diseases and the International Liver Transplantation Society.

[b26] Lodge JP, Jonas S, Oussoultzoglou E (2005b). Recombinant coagulation factor VIIa in major liver resection: a randomized, placebo-controlled, double-blind clinical trial. Anesthesiology.

[b27] Logan AC, Yank V, Stafford RS (2011). Off-label use of recombinant factor VIIa in U.S. hospitals: analysis of hospital records. Annals of Internal Medicine.

[b28] Ma B, Wang ZN, Zhang BR, Xu ZY, Yang LX, Chen KB, Li J (2006). Effect of recombinant activated factor VIIa on early recovery of patients undergoing cardiac valve replacement under cardiopulmonary bypass: a randomized double-blind placebo-controlled trial. Academic Journal of Second Military Medical University.

[b29] Mayer SA, Brun NC, Begtrup K (2005a). Recombinant activated factor VII for acute intracerebral hemorrhage. The New England Journal of Medicine.

[b30] Mayer SA, Brun NC, Broderick J, Davis S, Diringer MN, Skolnick BE, Steiner T, Europe/AustralAsia NovoSeven ICH Trial Investigators (2005b). Safety and feasibility of recombinant factor VIIa for acute intracerebral hemorrhage. Stroke: A Journal of Cerebral Circulation.

[b31] Mayer SA, Brun NC, Begtrup K (2008). Efficacy and safety of recombinant activated factor VII for acute intracerebral hemorrhage. The New England Journal of Medicine.

[b32] Mayer SA, Brun NC, Broderick J, Davis SM, Diringer MN, Skolnick BE, Steiner T, United States NovoSeven ICH Trial Investigators (2006). Recombinant activated factor VII for acute intracerebral hemorrhage: US phase IIA trial. Neurocritical Care.

[b33] Meng ZH, Wolberg AS, Monroe DM, Hoffman M (2003). The effect of temperature and pH on the activity of factor VIIa: implications for the efficacy of high-dose factor VIIa in hypothermic and acidotic patients. The Journal of Trauma.

[b34] Moltzan CJ, Anderson DA, Callum J (2008). The evidence for the use of recombinant factor VIIa in massive bleeding: development of a transfusion policy framework. Transfusion Medicine (Oxford, England).

[b35] Narayan RK, Maas AI, Marshall LF, Servadei F, Skolnick BE, Tillinger MN, rFVIIa Traumatic ICH Study Group (2008). Recombinant factor VIIA in traumatic intracerebral hemorrhage: results of a dose-escalation clinical trial. Neurosurgery.

[b36] O'Connell KA, Wood JJ, Wise RP, Lozier JN, Braun MM (2006). Thromboembolic adverse events after use of recombinant human coagulation factor VIIa. JAMA: The Journal of the American Medical Association.

[b37] Phillips LE, Zatta A, Kandane-Rathnayake R, Aoki N, on behalf of the Haemostasis Registry Steering Committee (2011).

[b38] Pihusch M, Bacigalupo A, Szer J, von Depka Prondzinski M, Gaspar-Blaudschun B, Hyveled L, Brenner B, F7BMT-1360 Trial Investigators (2005). Recombinant activated factor VII in treatment of bleeding complications following hematopoietic stem cell transplantation. Journal of Thrombosis and Haemostasis: JTH.

[b39] Planinsic RM, van der Meer J, Testa G (2005). Safety and efficacy of a single bolus administration of recombinant factor VIIa in liver transplantation due to chronic liver disease. Liver Transplantation: Official Publication of the American Association for the Study of Liver Diseases and the International Liver Transplantation Society.

[b40] Pugliese F, Ruberto F, Summonti D (2007). Activated recombinant factor VII in orthotopic liver transplantation. Transplantation Proceedings.

[b41] Raobaikady R, Redman J, Ball JA, Maloney G, Grounds RM (2005). Use of activated recombinant coagulation factor VII in patients undergoing reconstruction surgery for traumatic fracture of pelvis or pelvis and acetabulum: a double-blind, randomized, placebo-controlled trial. British Journal of Anaesthesia.

[b42] Sachs B, Delacy D, Green J (2007). Recombinant activated factor VII in spinal surgery: a multicenter, randomized, double-blind, placebo-controlled, dose-escalation trial. Spine.

[b43] Shao YF, Yang JM, Chau GY, Sirivatanauksorn Y, Zhong SX, Erhardtsen E, Nivatvongs S, Lee PH (2006). Safety and hemostatic effect of recombinant activated factor VII in cirrhotic patients undergoing partial hepatectomy: a multicenter, randomized, double-blind, placebo-controlled trial. American Journal of Surgery.

[b44] Simpson E, Lin Y, Stanworth S, Birchall J, Doree C, Hyde C (2012).

[b45] Yank V, Tuohy CV, Logan AC (2011). Systematic review: benefits and harms of in-hospital use of recombinant factor VIIa for off-label indications. Annals of Internal Medicine.

